# Injectable Nanocomposite Implants Reduce ROS Accumulation and Improve Heart Function after Infarction

**DOI:** 10.1002/advs.202102919

**Published:** 2021-10-31

**Authors:** Malka Shilo, Hadas Oved, Lior Wertheim, Idan Gal, Nadav Noor, Ori Green, Ester‐Sapir Baruch, Doron Shabat, Assaf Shapira, Tal Dvir

**Affiliations:** ^1^ The Shmunis School of Biomedicine and Cancer Research Faculty of Life Sciences Tel Aviv University Tel Aviv 6997801 Israel; ^2^ School of Chemistry Faculty of Exact Sciences Tel Aviv University Tel Aviv 6997801 Israel; ^3^ The Center for Nanoscience and Nanotechnology Tel Aviv University Tel Aviv 6997801 Israel; ^4^ The Department of Biomedical Engineering Faculty of Engineering Tel Aviv University Tel Aviv 6997801 Israel; ^5^ Sagol Center for Regenerative Biotechnology Tel Aviv University Tel Aviv 6997801 Israel

**Keywords:** cardiac tissue engineering, gold nanoparticles, heart disease, hydrogel, myocardial infarction

## Abstract

In a myocardial infarction, blood supply to the left ventricle is abrogated due to blockage of one of the coronary arteries, leading to ischemia, which further triggers the generation of reactive oxygen species (ROS). These sequential processes eventually lead to the death of contractile cells and affect the integrity of blood vessels, resulting in the formation of scar tissue. A new heart therapy comprised of cardiac implants encapsulated within an injectable extracellular matrix‐gold nanoparticle composite hydrogel is reported. The particles on the collagenous fibers within the hydrogel promote fast transfer of electrical signal between cardiac cells, leading to the functional assembly of the cardiac implants. The composite hydrogel is shown to absorb reactive oxygen species in vitro and in vivo in mice ischemia reperfusion model. The reduction in ROS levels preserve cardiac tissue morphology and blood vessel integrity, reduce the scar size and the inflammatory response, and significantly prevent the deterioration of heart function.

## Graphical abstract

This study presents a new heart therapy comprised of cardiac implants encapsulated within an injectable extracellular matrix‐gold nanoparticle composite hydrogel in mice ischemia‐reperfusion model. The particles promote the fast transfer of electrical signal between cardiac cells, whereas the composite hydrogel can absorb reactive oxygen species. The reduction in reactive oxygen species levels can preserve cardiac tissue morphology and functionality.

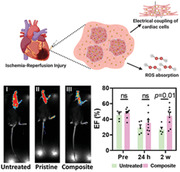

## 1 Introduction

Cardiovascular diseases account for 34% of all deaths in the United States with costs in excess of $500 billion annually.^[^ ^1^ ^]^ Myocardial infarction (MI) account for a significant segment of this population and is associated with sudden death, as well significant morbidity and mortality.^[^ ^1^, ^2^ ^]^ In MI, blood supply to the left ventricle is stopped due to a blockage in one of the coronary arteries, starving cells of oxygen. The lack of oxygen leads to ischemia, which further triggers the generation of reactive oxygen species (ROS), which are harmful to downstream cells, especially after reperfusion.^[^ ^3^, ^4^, ^5^, ^6^, ^7^, ^8^, ^9^ ^]^ These sequential processes eventually lead to the death of contractile cells and affect the integrity of blood vessels, leading to the formation of scar tissue.^[^ ^5^, ^6^, ^7^ ^]^ Since cardiomyocytes (cardiac muscle cells) cannot proliferate and the number of stem cells in the heart is limited, the heart has no regenerative capabilities, leading to chronic cardiac dysfunction. Complications following an initial MI include heart failure, recurrent ischemia and arrhythmias, which together cause a 5 year mortality rate of nearly 50%.^[^ ^10^ ^]^ Currently, the main solutions for end‐stage patients are heart transplantation and left ventricular assist devices (LVAD) therapy. As heart donors are scarce and since LVAD therapy may cause unpredicted complications and relatively short life time,^[^ ^11^ ^]^ it is essential to develop new strategies to stop progressive damage and promote regeneration.^[^ ^12^, ^13^, ^14^ ^]^

Cardiac tissue engineering has evolved as an interdisciplinary technological field combining principles from materials engineering and life sciences, as well as cardiology, with the goal of developing functional substitutes for the injured myocardium.^[^ ^15^, ^16^ ^]^ The fabrication of functional cardiac patches requires efficient organization of cells into an engineered tissue with morphological and physiological features resembling those of the natural tissue.^[^ ^15^, ^17^, ^18^ ^]^ This task is highly challenging as the signaling factors that promote cardiac tissue assembly have not yet been completely identified. In vivo, this process synergistically involves two main components, namely cardiomyocytes, the contracting cells of the heart, and the extracellular matrix (ECM), which provides structural and functional support.^[^ ^15^, ^17^ ^]^ Therefore, when employing a tissue engineering approach, rather than simply injecting cells into the diseased area to repopulate the injured heart and restore function, the cells are seeded in 3D biomaterials prior to transplantation.^[^ ^17^ ^]^ These materials serve as scaffolds that mimic the ECM and encourage cell reorganization into a functional heart patch.^[^ ^15^, ^17^ ^]^

Over the past years, several groups, including ours, have developed various biomaterial scaffolds for cardiac tissue engineering, identifying key factors in the 3D scaffold, as well as external cues that promote the assembly of functional cardiac patches.^[^ ^15^, ^19^, ^20^, ^21^, ^22^, ^23^, ^24^, ^25^, ^26^, ^27^, ^28^, ^29^ ^]^ However, several key obstacles still hamper the advancement of this technology. First, it is extremely difficult to control electrical coupling between adjacent cardiomyocytes. After isolation and seeding within the biomaterials, the cells become rounded, and over time form immature electrical interactions. Therefore, inducing rapid reformation of mature electrical interactions is critical for the success of the cardiac patch. Additionally, it is critical to ensure proper electrical integration between the engineered patch and the host myocardium. Recently, our group and others have reported that conductive nanostructures can be designed to interact with excitable cells to generate electronic interfaces,^[^ ^30^, ^31^, ^32^ ^]^ enhance cellular excitability,^[^ ^33^, ^34^, ^35^, ^36^, ^37^ ^]^ and even create electrical shortcuts.^[^ ^17^, ^36^ ^]^ We have shown that gold nanowires, nanospheres, and nanorods can serve as synthetic couplers between cardiac cells, enhancing the expression of connexin 43 electrical coupling proteins, and overall improving the electrical signal transfer between adjacent cell bundles.^[^ ^25^, ^27^, ^29^, ^38^ ^]^ However, when aiming at heart regeneration after MI, introducing a cardiac patch into the left ventricle may not be efficient without first eliminating the existing stress factors, such as ROS, which are produced during the injury. At the reperfusion, when the oxygen molecules are suddenly returned to the ischemic tissue, a unique injury response occurs, resulting in an imbalance between the rate of ROS generation and the ability of the tissue to detoxify these molecules. The damage from these factors is progressive, affecting the implanted tissue and jeopardizing the success of the treatment.^[^ ^6^, ^39^, ^40^, ^41^, ^42^, ^43^, ^44^ ^]^

**Figure 1:**
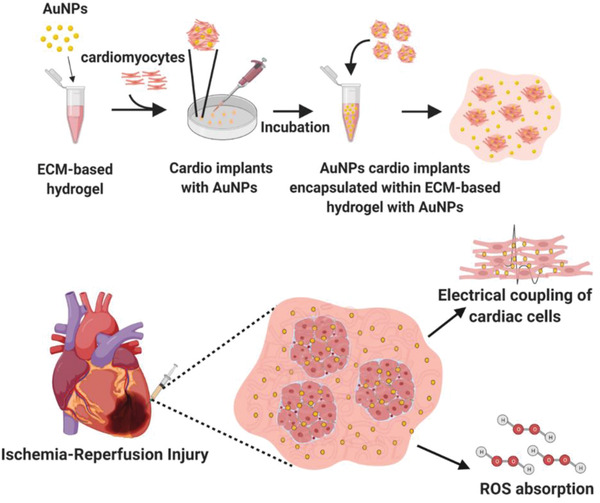
Schematic illustration of the study outline. Top: fabrication of the ECM‐AuNPs nanocomposites. Bottom: the treatment provided by the newly designed cellular nanocomposite.

Here, we developed a new regenerative approach to promote the efficient transfer of the electrical signal between cells, reduce ROS levels and regenerate the diseased heart. Gold nanoparticles (AuNPs) were incorporated into an ECM‐based hydrogel (composite hydrogel), which served as a scaffolding material for cardiac cells, and which together formed microcardiac implants with fast conduction velocity. Multiple cardiac implants were then encapsulated in the composite hydrogel at a lower concentration to form an injectable tissue engineering system. Following injection to a mouse model of ischemia reperfusion, the entire complex absorbed ROS, reducing their levels and protecting the cellular implants from a harsh microenvironment; reduced scar size; and prevented the deterioration of heart function (**Figure** **1**).

## 2 Results and Discussion

**Figure 2:**
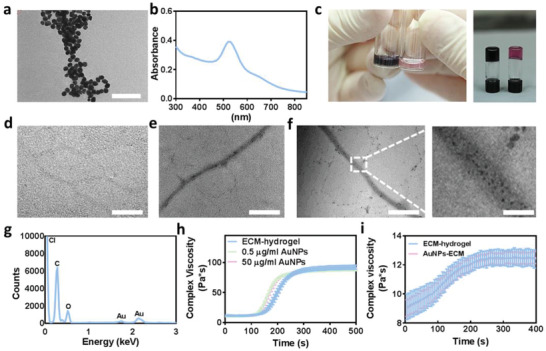
ECM‐AuNPs composite characterization. a) TEM image of the AuNPs, scale bar 200 nm. b) Absorbance spectrum of the AuNPs. c) Image of pristine hydrogel (right) compared to composite hydrogel (left), before (left panel) and after (right panel) solidification. d–f) TEM section images of d) pristine hydrogel, e) low AuNP concentration composite hydrogel and f) high AuNP concentration composite hydrogel, scale bar 1 µm. g) EDX analysis of the composite hydrogel. h,i) Rheological properties of the composite hydrogel containing h) 0.6% w/v and i) 0.2% hydrogel.

Spherical AuNPs were synthesized as previously described.^[^ ^45^ ^]^ The size, shape, and uniformity were measured using transmission electron microscopy (TEM) and dynamic light scattering (DLS). The particles were monodispersed in size with an average diameter of 24 ± 3 nm and a zeta potential of −36 ± 1.5 mV (**Figure** **2a**; and Figure S1, Supporting Information). UV–vis spectrum revealed a strong absorption at 520 nm (Figure 2b). To form the composite hydrogel, the particles were encapsulated within the pristine hydrogel, mixed, and allowed to form electrostatic interactions between the negatively charged groups of the AuNPs and the amine groups within the polymeric backbone.^[^ ^46^, ^47^, ^48^ ^]^ Both the pristine and composite solutions were reverse thermoresponsive and formed a gel after heating to 37 ^°^C (Figure 2c). As shown by TEM micrographs, pristine hydrogel fibers were almost undetectable (Figure 2d), while the fibers within the composite hydrogel were easily detected due to the integrated AuNPs (Figure 2e,f). Furthermore, the particles were homogenously dispersed on the collagen fibers. Energy‐dispersive X‐ray spectroscopy (EDX) analysis confirmed the presence of AuNPs on the hydrogel fibers (Figure 2g). Rheological measurements were performed, allowing to confirm that the presence of the particles did not affect the mechanical properties of the pristine hydrogel (Figure 2h,i).

**Figure 3:**
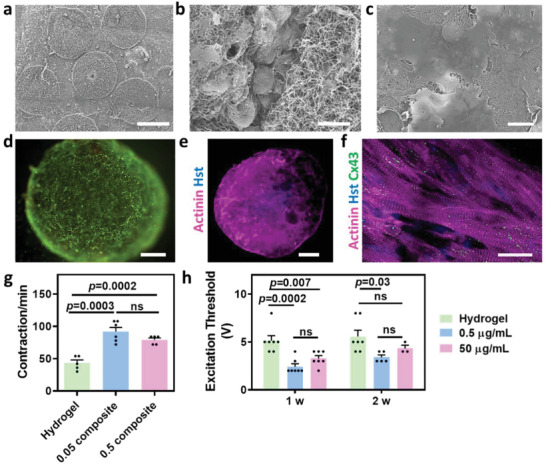
Cardiac implant organization and function. a) SEM image of the entire complex, scale bar 600 µm. b) SEM image of the cellular implants 1 day after cell encapsulation and the surrounding composite hydrogel, scale bar 10 µm. c) SEM image of the low AuNP concentration composite hydrogel 1 week after cell encapsulation, scale bar 10 µm. d) Viability test for the low AuNP concentration composite hydrogel 1 week after cell encapsulation (live cells in green and dead cells in red), scale bar 100 µm. e,f) Cardiac sarcomeric actinin immunostaining of low AuNP concentration composite hydrogel on day 14 after cell encapsulation. e) Low magnification. Actinin in pink, nuclei in blue, scale bar 500 µm. f) High magnification. Actinin in pink, connexin 43 in green and nuclei in blue, scale bar 20 µm. g) Contraction rate of the pristine (*n* = 5) and composite hydrogels 2 weeks (*n* = 6,5) after cell encapsulation. h) Excitation threshold of the pristine and composite hydrogels, 1 and 2 weeks after cell encapsulation (*n* > 3).

Next, we sought to form the cellular part of the cardiac patch. As a proof of concept, cardiac cells were isolated from the left ventricles of neonatal rats as previously described.^[^ ^49^ ^]^ The cells were encapsulated within 0.6% w/v pristine hydrogel in its low viscosity state, containing different AuNP concentrations. Subsequently, multiple 2 µL droplets were placed in a petri dish and incubated at 37 ^°^C to achieve gelation. These cardiac microimplants were further encapsulated within a 0.2% w/v composite hydrogel. In this setup, the cells can form electromechanical interactions and form a microtissue within the droplet.^[^ ^26^ ^]^ This system can be easily injected through a syringe without compromising the integrity of the engineered tissue and without jeopardizing the established cell‐cell interactions. Scanning electron microscopy (SEM) analysis of the complex (**Figure** **3a**,b) showed the highly dense, fibrous structure of the ECM within the cellular implants and the surrounding lower density fibers. As shown, on the first day, the cells within the implants were still round, forming interactions with the fibers (Figure 3b). Seven‐days postencapsulation, the cells were able to spread in the hydrogel's fibrous network and on its surface (Figure 3c). Cell viability within the composite hydrogel was assessed by a live/dead assay, which revealed high viability within composite hydrogels containing both low and high AuNP concentrations (Figure 3d; and Figure S2, Supporting Information).

We next sought to evaluate the effect of AuNPs on the morphology and function of engineered cardiac implants. As shown, 2 weeks after cell encapsulation, the cells within the entire composite hydrogel droplet expressed high levels of sarcomeric actinin (Figure 3e). Higher magnification images revealed aligned, elongated cells with massive striation, as indicated by the actinin expression (Figure 3f). Moreover, based on the pronounced connexin 43 staining, the cells were electrically coupled to each other (Figure 3f). This morphology and protein expression pattern further support the notion that the composite hydrogel did not have a negative effect on the encapsulated cells.

We have previously shown that AuNPs can increase the electrical signal within engineered tissues in macroporous or electrospun fiber scaffolds.^[^ ^25^, ^27^ ^]^ Here, we sought to evaluate this effect within the composite hydrogel, which has an additional advantage in that it can be injected into the desired site, rather than transplanted. As shown, the contraction rate of the composite hydrogel‐based implants was significantly higher than that of the pristine hydrogel‐based implants (Figure 3g). Next, pristine and composite hydrogel‐based implants were subjected to an external electrical field. The minimum voltage which was required to induce synchronous contraction of the entire implant at a frequency of 1 and 2 Hz was defined as the excitation threshold. As shown, 1 and 2 weeks postencapsulation, the composite hydrogel‐based implants had a lower excitation threshold (Figure 3h; and Movies S1–S3, Supporting Information). Taken together, these results indicate that the cells within the pristine hydrogel can interact with the AuNPs.

**Figure 4:**
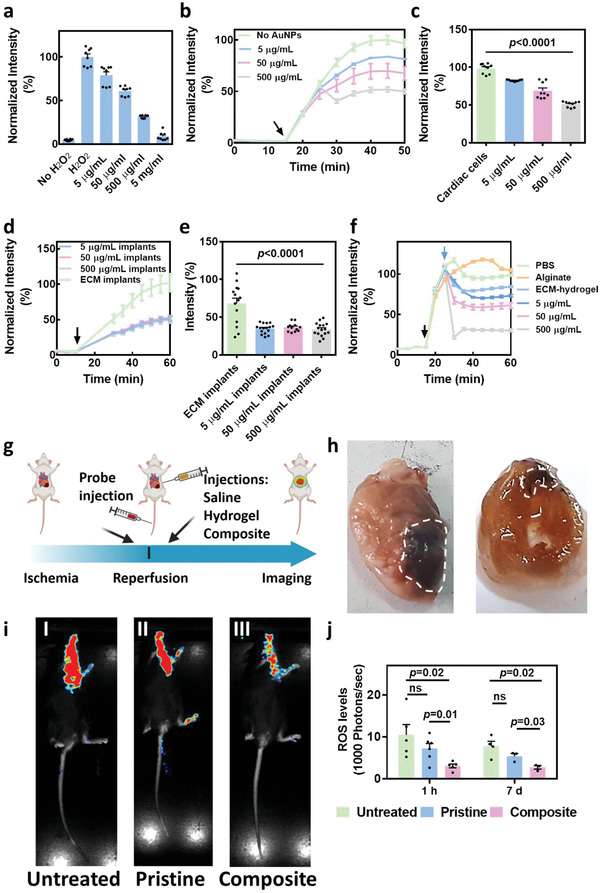
Effect of the composite hydrogel on ROS levels a–f) in vitro. a) ROS levels following incubation of different concentration of AuNPs with H_2_O_2_ (*n* = 8). (b) ROS levels following incubation of different concentration of AuNPs with cardiac cells (black arrow indicates menadione addition) (*n* = 9). c) ROS levels 20 min after menadione addition. d) ROS levels following the encapsulation of AuNPs at different concentrations within cardiac implants (black arrow indicates menadione addition). e) ROS levels 20 min after menadione addition (*n* ≥12). f) ROS levels following the addition of different treatments to cover the cardiac cells – PBS as a control, alginate, pristine hydrogel, and different concentrations of AuNPs encapsulated within pristine hydrogel (black arrow indicates menadione addition, blue arrow indicates treatment addition). g–j) Effect of the composite hydrogel in vivo after ischemia‐reperfusion injury (IRI). g) Schematic representation of the in vivo study, h) Macroimage of the hearts treated with the composite hydrogel and harvested 6‐weeks post‐IRI. White dashed areas indicate the treatment region. i) Bioluminescence imaging of (I) untreated, (II) pristine hydrogel‐, and (III) composite hydrogel‐treated mice, 1 h post‐IRI. j) Quantification of ROS levels following the different treatments 1 h (*n* ≥ 5) and 1 week post‐IRI (*n* ≥ 3).

The presence of ROS within an injured tissue leads to expansion of cell death and to deterioration of the diseased organ.^[^ ^6^, ^40^, ^41^ ^]^ Such pathophysiology may jeopardize the success of any cell therapy, as the cells are subjected to a toxic environment. Therefore, we sought to test our hypothesis that the composite hydrogel can serve not only as a scaffolding material for cells, but also as a ROS scavenger. To assess ROS concentration ex vivo, we used a known ROS‐chemiluminescence probe showing light emission upon chemical interaction with H_2_O_2_ and other ROS derivatives.^[^ ^50^ ^]^ First, we tested the effect of free AuNPs on ROS levels in the presence of H_2_O_2_. AuNPs at different concentrations were incubated with H_2_O_2_, and ROS levels were measured. As shown, ROS levels were inversely correlated to AuNP concentration (**Figure** **4a**). We then tested the effect of AuNPs on ROS levels produced by cardiac cells. Cardiac cells were seeded in a 24‐well plate. Five days later, intracellular ROS levels were increased by incubation with an oxidative injury‐inducing agent (menadoine; Figure 4b, black arrow). Subsequently, different AuNP concentrations were added to the culture. As shown, the presence of AuNPs resulted in a decrease in ROS levels produced by the cells (Figure 4b,c).

When cardiac patches or dissociated cells are transplanted or injected into a diseased microenvironment, such as the infarcted heart, stress may be induced followed by ROS secretion. Therefore, we next sought to examine the ability of the composite hydrogel to decrease ROS levels produced by the cells within the same implants. 7 days old implants were incubated with menadione for the production of ROS (Figure 4d, black arrow), and the intracellular ROS levels were measured (Figure 4d,e). As shown, all AuNP concentrations within the composite hydrogel‐based implants were able to significantly reduce ROS levels. No significant differences were detected between implants with various AuNP concentrations. This may be attributed to the fact that the AuNPs were bound to the matrix by electrostatic interaction and were not internalized by the cells.

We next simulated the effect of composite hydrogel injection into the diseased heart, where the cells in situ already produce ROS (Figure 4f). Cardiac cells were seeded on 2D surfaces and the intracellular ROS levels were increased by menadione (black arrow). ROS levels were followed for 20 minutes, and then either the composite hydrogels containing different AuNP concentrations, or an alginate hydrogel (as a control), were added to the cell cultures to form a thin layer covering the cells (blue arrow). As another control, phosphate buffered saline (PBS) was added to the cultures at the same volume as the supplemented hydrogels to evaluate the effect of ROS dilution by hydrogel addition. As shown, the pristine hydrogel itself had a slight protective effect on cardiac cells (Figure 4f; and Figure S3, Supporting Information). However, the composite hydrogels were able to further decrease ROS levels (Figure 4f). In contrast, commercial alginate had no effect, and ROS levels continued to increase. The addition of PBS to control cultures led to a temporary reduction of ROS levels due to medium dilution, though ROS levels rapidly increased again several minutes later. A slight decrease in ROS levels was observed after treatment with the composite hydrogel with a higher concentration of AuNPs (Figure 4b,f). Although this effect is temporary and not significant, it needs to be further investigated. Overall, these in vitro results emphasize the importance of the composite hydrogel not only for increasing electrical signaling between cardiac cells, but also for decreasing ROS levels.

In order to evaluate the effect of the composite hydrogel on ROS accumulation, and thereby on the diseased heart, in vivo, we performed an ischemia‐reperfusion injury in mice (Figure 4g). This model simulates the damage to the left ventricle after a heart attack when the blocked blood vessels are reopened. The animals were anesthetized, and their major coronary artery was blocked for 45 min. Five min before reperfusion, a ROS‐specific luminescent probe was intravenously injected, and the composite hydrogel, pristine hydrogel, or saline was injected directly into the infracted area. As shown in the macropictures, the composite hydrogel accumulated within the left ventricle of the injured heart (Figure 4h). ROS‐level measurements 1 hour postreperfusion, by whole animal bioluminescence imaging (Figure 4i), revealed that the composite hydrogel significantly reduced ROS levels in comparison to the other groups (Figure 4i,j). Since ROS secretion by the injured cells within the diseased heart is progressive and continuous, we imaged the same mice again 1‐week post‐treatment, and ROS levels were assessed, revealing a continued effect of the composite (Figure 4j; and Figure S4, Supporting Information). No significant difference was observed between mice injected with saline and those injected with the pristine hydrogel at either time point.

**Figure 5:**
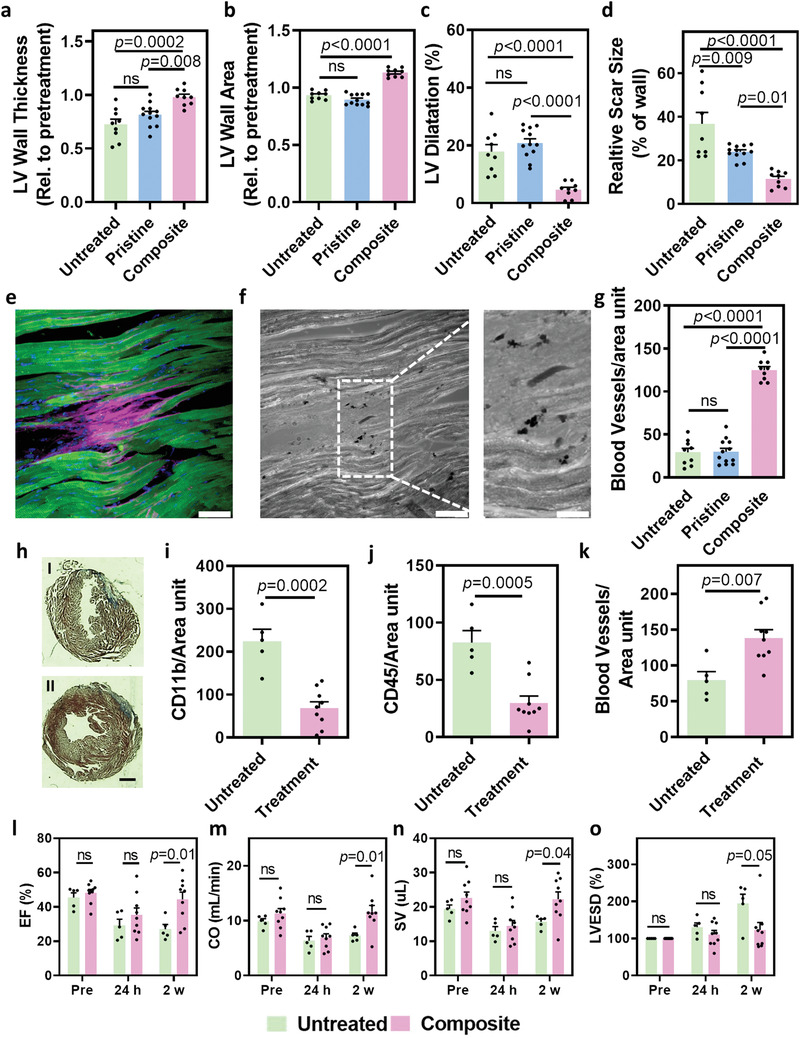
Effect of the composite hydrogel on structure, composition and function of the diseased heart. a–g) Effect of the cell‐free composite hydrogel on the structure of the heart (*n* = 9). a–d) Quantification of heart morphology based on MRI sections. a) LV wall thickness, b) LV wall area, and c) LV dilatation, 6 weeks post‐IRI. d) Relative scar size as indicated by Masson's trichrome histology 6‐weeks post‐IRI. e) Fluorescent immunostaining of the scar tissue 6 weeks post‐IRI for the composite‐hydrogel treatment. Cardiac troponin is in green, collagen in pink, and nuclei in blue, scale bar 20 µm. f) Bright‐field image of the scar tissue presented in e). AuNPs can be clearly observed as small black dots, scale bar 20 µm. g) Quantification of number of blood vessels within the left ventricle following all treatments. h–o) Effect of the cellular implant‐containing composite hydrogel on the structure, composition, and function of the heart. h) Masson's trichrome histology of sections of the untreated (I) and treated (II) heart. Scale bar 1.5 mm. i) Quantification of macrophages in the left ventricle. j) Quantification of leukocytes in the left ventricle. k) Quantification of number of blood vessels within the left ventricle. l–o) Heart function after treatment with the cellular implant‐containing complex hydrogel. l) Ejection fraction (EF). m) Cardiac output (CO). n) Stroke volume (SV). o) LV end‐systolic volume (LVESD) (*n* = 5 for untreated and *n* = 9 for treated).

We next sought to assess the effect of the different treatments on the heart structure. As we wanted to isolate the effect of ROS reduction by the composite hydrogel, at this stage the implant did not contain any cells. Magnetic resonance imaging (MRI) was performed prior to treatment (baseline), and 6 weeks post‐treatment (Figure S5, Supporting Information). Data analyses of MRI images revealed that both the thickness and area of the left ventricular (LV) wall of the composite‐treated animals were significantly larger than in the untreated group, indicating less injury to the wall (**Figure** **5a**,b). Furthermore, significantly less dilation of the LV could be observed in the composite hydrogel group (Figure 5c). To further support these results, at that point the animals were sacrificed, and their hearts were fixed, sliced, and stained for Masson's trichrome (Figure S6, Supporting Information), and morphometric analyses were performed. As shown, the relative scar size was significantly smaller in the composite group when compared to either pristine or untreated animals (Figure 5d). Overall, such reduced damage and preservation of LV wall dimensions may assist in preservation of heart function.^[^ ^51^, ^52^, ^53^, ^54^, ^55^, ^56^ ^]^

To determine whether the ability to absorb ROS in the LV after injury contributes to protection of the cells, we analyzed the integrity of the LV tissue downstream of the injury site. As shown, compared to the control groups, cardiomyocytes within the LV of animals treated with the composite hydrogel were elongated and aligned, as evidenced by the massive, organized troponin T expression (Figure 5e,f; and Figure S7, Supporting Information). The AuNPs were arranged in small clusters (Figure 5f, black dots) and could be observed in‐between cardiac cell bundles within collagen islands (Figure 5e, pink). The dispersion of AuNPs in close proximity to healthy cardiomyocytes in the composite hydrogel animals and the lack of ordered cells in the control groups lacking AuNPs, may suggest the beneficial, cardioprotective effect of the composite hydrogel.

An important component of the LV is a well‐organized blood vessel network. This network nourishes the myocardium, supplying oxygen, and nutrients.^[^ ^57^, ^58^, ^59^ ^]^ Under ischemic conditions, blood vessel‐forming cells are deprived of oxygen, and lumen structure is destroyed. Upon opening the blocked coronary vessels and resupplying the necessary nutrients, fluxes of ROS further harm the cells, leading to progressive, irreversible damage.^[^ ^6^, ^40^, ^41^, ^42^ ^]^ Therefore, we next sought to analyze the blood vessels downstream of the blocked area. As shown, immunostaining for CD31 within the diseased area revealed a higher density of blood vessels in mice treated with the composite hydrogel (Figure S8, Supporting Information). Further image analysis revealed this difference in blood vessel density to be significant (Figure 5g). Based on our experiments and other studies,^[^ ^60^, ^61^ ^]^ we suggest that an exchange interaction occurs between the unpaired electrons of the ROS and the conduction‐band electrons of the AuNPs. That interaction allows the AuNPs to absorb and eliminate the ROS, and thereby reduce the oxidative damage within the heart tissue following myocardial ischemia‐reperfusion injury. However, further studies should be performed to determine the exact mechanism of ROS absorption. Next, we sought to evaluate the effect of the whole complex, including the cardiac implants within the composite ECM, on the deterioration of the LV. Ischemia‐reperfusion surgery was performed as described above, and just prior to reperfusion, the cellular composite complex or saline was injected directly into the infarcted area. Two weeks after injection, the LV structure, composition and function were assessed. As shown by Masson's trichrome staining, the LV wall of the untreated animals became thinner with strong blue staining, indicating the formation of collagenous scar tissue (Figure 5h, I; and Figure S9, Supporting Information). In contrast, the LV wall of the treated animals was only slightly affected, revealing normal wall structure (Figure 5h, II; and Figure S9, Supporting Information).

We next sought to evaluate inflammation within the LV. The role of immune cells in the injured heart is still not fully clear and may have both negative and positive effects.^[^ ^62^, ^63^ ^]^ In any case, the immune system responds to a pathology, such as that occurring from cell death due to ROS accumulation. We hypothesized that reduced levels of ROS, due to their absorbance by the composite hydrogel, would lead to decreased injury and therefore to reduced inflammation. Therefore, the LV wall was stained for macrophages, which clear apoptotic cells, and leukocytes, which may play a role in healing after injury. As shown by CD11b and CD45 staining, both markers were found in significantly lower levels within the treated LV (Figure 5i,j; and Figures S10 and S11, Supporting Information). Furthermore, analysis of the LV revealed a significantly higher density of blood vessels in the treated mice in comparison to the untreated (Figure 5k; and Figure S12, Supporting Information). Furthermore, cells expressing both troponin and rat major histocompatibility complex (MHC) class I could be detected in‐between mouse cardiac cell bundles, indicating on the existence of the injected cell implants at the end of the experiment (Figure S13, Supporting Information). Finally, after analyzing the structure and composition of the LV after treatment, we sought to evaluate its function by echocardiography. Functional measurements were performed preinjury, 1 day after injury, and 2 weeks after injury. We hypothesized that the injury itself would temporarily affect heart function after 1 day. However, since high ROS accumulation would be prevented by the composite hydrogel, further deterioration of LV function would be avoided. As shown, in contrast to untreated mice, all measurements, including ejection fraction (EF), cardiac output (CO), stroke volume (SV), and LV end‐systolic volume (LVESD) reached their baseline levels 2 weeks post‐treatment, indicating the efficacy of the treatment (Figure 5l–o; and Figures S14 and S15, Supporting Information).

## 3 Conclusion

In summary, a new, cellular, ECM‐AuNP‐based composite hydrogel that may provide heart therapy, was demonstrated. First, we have shown the ability of the injectable composite hydrogel to support the formation of an engineered cardiac tissue with aligned and elongated cardiac cells exhibiting massive striation and expressing electrical coupling proteins. Usually, injectable systems are used to introduce dissociated cells into a defective tissue. While the cells engraft and form interactions with the host, they also need to assemble into a functioning tissue, which is a time‐ and energy‐consuming process, occurring under harsh conditions. Here, the ability to form microtissue with the hallmarks of mature tissue and fast electrical‐signal propagation, prior to injection, may be an advantage in regenerative medicine. Second, the potential of the composite hydrogel to absorb ROS in an ischemia‐reperfusion model was demonstrated. As the presence of ROS within the LV expands the injury, leading to extensive cell death,^[^ ^6^, ^39^, ^40^, ^41^, ^42^ ^]^ inflammation^[^ ^41^, ^64^, ^65^ ^]^ and, overall, to progressive damage, the ability to eliminate ROS may be extremely beneficial for treating MI and other heart diseases, as well as other injuries in different organs. Our results indicate that injection of the composite complex leads to cardioprotection within the LV, which, in turn, leads to the preservation of heart structure and function. Moreover, blood vessels within the LV were maintained, and inflammatory components were reduced, indicating a less severe injury. We envision that the future use of Induced pluripotent stem cell (iPSC)‐derived cardiomyocytes with an ECM‐based composite hydrogel^[^ ^20^ ^]^ that can eliminate ROS from the diseased area may significantly advance the tissue engineering and regenerative medicine fields. It is important to note that this proof of concept study was designed to assess the ability of the composite hydrogel‐based implants to reduce ROS levels in an animal model and at a timeline, in which the damage is maximal. This experimental timeline, where the treatment is given prior to the opening of the occulated blood vessel may not be ideal for the clinic, where the initial urgent focus should be given to recanalization of the blocked artery. Therefore, future studies, in larger animals should be conducted to determine the therapeutic effect after intracardiac delivery of the implant as a secondary treatment.

## 4 Experimental Section

### AuNPs Synthesis

Spherical AuNPs were synthesized using sodium citrate according to the methodology described by Enüstun and Turkevich.^[^ ^45^ ^]^ Briefly, 41.4 µL of 50% w/v HAuCl4 (Sigma‐Aldrich) mixed with 20 mL purified water was used. This mixture was heated until boiling, and then 404 µL tri‐sodium citrate (Merck) were added under stirring. Within a few minutes, the color of the solution changed from clear yellow to deep red. The solution was cooled to room temperature and centrifuged at 12 000 RPM for 20 min until the nanoparticles precipitated and a clear suspension was obtained. The final concentration of 5 mg mL^−1^ was measured using Flame Atomic Absorption Spectroscopy (FAAA, SpectrAA 140, Agilent Technologies). Nanoparticles were characterized using a Spectrophotometer, TEM and EDX (Supporting Information).

### ECM‐Based Hydrogel Preparation

Omenta were decellularized as previously described.^[^ ^20^ ^]^ The final concentration of decellularized omentum was 1% w/v (Supporting Information).

### AuNPs Implants Preparation

AuNPs at a concentration of 0.05 and 0.0005 mg mL^−1^ were synthesized. Then, AuNPs were encapsulated within 1% w/v pristine hydrogel at a 1:4 AuNPs:hydrogel ratio in order to achieve a final hydrogel concentration of 0.8%. For control implants, the pristine hydrogel was diluted with 5% FBS – M199 medium at the same ratio. Rheological properties of the different implants were tested as described in the Supporting Information.

### Cardiac Cell Isolation and Seeding

Neonatal Sprague‐Dawley rat ventricular cardiomyocytes were isolated using established protocols^[^ ^49^ ^]^ and cultured within pristine hydrogels or cell culture plates for luminescence readings (Supporting Information).

### Excitation Threshold

Excitation threshold was defined as the voltage at which the cellular implants started to contract synchronously at frequencies of 1 and 2 Hz. Cellular implants were placed in M199 medium at 37 °C between two carbon electrode rods placed 1 cm apart in a petri dish. Implants were stimulated with 50 ms square pulses delivered at a rate of 1 or 2 Hz, starting with an amplitude of 1 V electrical field. The amplitude was increased in 1 V increments. Implants were imaged using an inverted fluorescence microscope (Nikon Eclipse TI). Movies were acquired with a Hamamatsu Orcaflash 4.0 (Hamamatsu) at 100 frames per second using NIS element software (Nikon).

### Ischemia‐Reperfusion Injury (IRI) Surgery

IRI was induced in C57BL/6 12 weeks old male mice. Mice were anesthetized with 3% isoflurene, intubated, and ventilated. The chest was opened by a left thoracotomy, the pericardium was removed, and the major coronary artery was occluded with 0–8 Prolene sutures (W2777, Ethicon) for 45 min. Then, 25 µL of treatment solution was injected directly onto the scar tissue (insulin syringe, 30G, BD Micro Fine Plus). Treatment groups were: saline as a control, pristine hydrogel and composite hydrogels including AuNPs or a 1:1 ratio of AuNPs and Gd‐AuNPs. All hydrogel samples were prepared to reach a final concentration of 0.2% hydrogel, at a 1:3 hydrogel: AuNPs ratio (0.5 mg mL^−1^) or medium. Samples were injected in their solution form at 4 °C. For the whole‐complex experiment, cardiac implants were engineered as described above, with 0.0005 mg mL^−1^ AuNPs. The implants were cultured for two weeks before encapsulation within the composite hydrogel. Treatment groups were: saline as a control and the cellular composite hydrogel (each animal received five cardiac implants encapsulated within a single 25 µL droplet of the composite hydrogel). The treatments were injected directly into the muscle by a 27G needle. Subsequently, coronary artery ligation was removed, and an ROS‐luminescence probe was intravenously injected. Mice were examined by bioluminescence imaging 1 h and 1‐week postsurgery. MRI imaging was performed before the surgery, as well as 1‐ and 6‐weeks postsurgery. For whole‐complex experiments, echocardiography measurements were performed before and 1‐ and 14‐days postsurgery. At the end of the experiment, mice were sacrificed, and their hearts were removed for further analysis.

### Bioluminescence Imaging

Mice were injected intravenously with 100 µL of ROS‐chemiluminescent luminophore and placed in a BioSpace Lab PhotonIMAGER (Bio Space Lab, France) under 1.5% isoflurane in pure oxygen. Imaging was performed 1 h postprobe injection and analyzed using M3Vision software. Mice were imaged again 1 week postsurgery.

### Assessments of (LV) Remodeling and Function

Transthoracic echocardiography was performed with a Vevo 2100 instrument (VisualSonics, Toronto, Ontario, Canada) equipped with an MS‐400 imaging transducer. Light anesthesia was induced by inhalation of 2% isoflurane. Isoflurane flow was controlled in order to maintain the heart rate at >400 bpm in all mice. Mice were fixed to an echocardiogram measuring platform heated to 37 ^°^C during the measurements. B‐mode and M‐mode tracings of the LV endocardial border in a parasternal long axis were conducted to directly evaluate heart measurements, including ejection fraction (EF), cardiac output (CO), stroke volume (SV), and LV end‐systolic volume (LVESD). All measurements were analyzed by Vevolab software and averaged for three cardiac cycles.

### Morphometric Analysis

For morphometric analyses, LV wall area, LV wall thickness (at least 12 measurements along the wall) and relative scar thickness (at least 10 measurements) were calculated 6 weeks post‐IRI for each mouse, relative to the same parameters from the 1 week imaging. All calculations were performed on the middle section, at EDV frame. In order to assess the results, hearts were fixed in 4% formaldehyde (24 h at 4 °C) and embedded in OCT. Sections (40 µm thick) were prepared using a Cryotome FSE cryostat (Thermo Scientific), affixed to X‐tra adhesive glass slides (Leica Biosystems, Wetzler, Germany), dehydrated in graduated ethanol steps (70–100%) and stained with Masson's trichrome. All sections were imaged and examined using a binocular microscope (NIKON SMZ18). The average LV wall thickness was calculated from at least ten measurements of wall thickness whereas relative scar size was determined from measurements of scar area relative to the entire LV wall area in each animal. All parameters were calculated using ImageJ (1.51n, Wayne Rasband, NIH USA).

### Statistical Analysis

Statistical analysis data are presented as mean ± sem. Differences between samples were measured using ANOVA analysis followed by post‐hoc Tukey test. Analyses were performed using GraphPad prism version 6.00 for windows (GraphPad Software). *p* < 0.05 was considered significant.

### Animal Study

All mice were treated according to ethical regulations of Tel Aviv University. Permission was granted by the Institutional Animal Care and Use Committee (IACUC) of Tel Aviv University, protocol number 04‐20‐012‐ “Treatment of biological hydrogel and cardiac cells on cardiac regeneration after ischemia reperfusion injury in mice model”.

## Conflict of Interest

The authors declare no conflict of interest.

## Author Contributions

M.S. and T.D. conceived the idea and designed the experiments. M.S. performed all experiments. H.O. performed cell culture experiments. I.G. and N.N. isolated cardiac cells. O.G. and D.S. synthesized the ROS probe. L.W. and E.S.B. synthesized and analyzed the NP composites. A.S., M.S., and T.D. analyzed data. M.S. and T.D wrote the manuscript. The study was directed by T.D.

## Supporting information

Supporting Information

Supplemental Movie 1

Supplemental Movie 2

Supplemental Movie 3

## Data Availability

Data available in article and Supporting Information.
