# Supplementary material for: Injectable Nanocomposite Implants Reduce ROS Accumulation and Improve Heart Function after Infarction
Source: Adv Sci (Weinh). 2021 Oct 31;8(24):2102919. doi: 10.1002/advs.202102919 (PMC8693049; doi:10.1002/advs.202102919)
Supplement: Supplementary file 1 — Supporting Information [file ADVS-8-2102919-s004.pdf]

© 2021 Wiley-VCH GmbH

ADVANCED SCIENCE
Open Access

Supporting Information

for *Adv. Sci.*, DOI: 10.1002/advs.202102919

Injectable nanocomposite implants reduce ROS accumulation and improve heart function after infarction

Malka Shilo[1], Hadas Oved[1], Lior Wertheim[1,2], Idan Gal[1], Nadav Noor[1,2], Ori Green[3], Ester-Sapir Baruch[1,2], Doron Shabat[3], Assaf Shapira[1], Tal Dvir[1,2,4,5] *

[1]The School for Molecular Cell Biology and Biotechnology, Faculty of Life Sciences, Tel Aviv University, Tel Aviv 6997801, Israel.

[2]School of Chemistry, Faculty of Exact Sciences, Tel Aviv University, Tel Aviv 6997801, Israel.

[3]The Center for Nanoscience and Nanotechnology, Tel Aviv University, Tel Aviv 6997801, Israel.

[4]Department of Biomedical Engineering, Faculty of Engineering, Tel Aviv University, Tel Aviv 6997801, Israel.

[5]Sagol Center for Regenerative Biotechnology, Tel Aviv University, Tel Aviv 6997801, Israel.

*Correspondence to Tal Dvir: tdvir@tauex.tau.ac.il

Methods

Scanning electron microscopy (SEM). Samples were mounted onto aluminum stubs with conductive paint and sputter-coated with an ultrathin (150 Å) layer of gold in a Polaron E 5100 coating apparatus (Quorum Technologies, Laughton, UK). The samples were viewed under JSM-840A SEM (JEOL, Tokyo, Japan). Higher resolution images were obtained using a JSM-6700F SEM (JEOL).

Energy-dispersive X-ray spectroscopy (EDX). AuNPs implants were imaged after mounting onto aluminum stubs with conductive paint and without additional coating using a Quanta 200 FEG Environmental Scanning Electron Microscope (ESEM) with a field-emission gun (FEG) electron source. Imaging was carried out under low vacuum with a high voltage of 20 kV and a working distance of 7.3 mm.

AuNPs characterization. The size, shape and uniformity of AuNPs were measured using transmission electron microscopy (TEM) (JEM-1400 Plus, JEOL). Samples were prepared by dropping 5 µL of AuNPs solution on a copper grid and then left to dry overnight before imaging. The absorption spectrum of AuNPs was evaluated using a UV-Vis Spectrophotometer (NanoDrop, Wilmington, DE). The size and charge of AuNPs were tested in Dynamic Light Scattering (DLS) and Zeta potential (Zetasizer, Malvern). For Gd-AuNPs, negative staining using uranyl acetate was used for AuNPs and Gd-AuNPs samples. Contrast images were acquired in TEM and captured using SIS Megaview III and iTEM (Olympus). Also, FTIR (Nicolet iS10 Mid Infrared FT-IR Spectrometer, Thermo Scientific) analysis was performed for all synthesis steps: AuNPs, DTPA complex, GdL complex and Gd-AuNPs.

ECM-based hydrogel preparation. Omenta were decellularized as previously described[1]. Briefly, omenta from healthy pigs (Kibbutz Lahav – designated for the food industry) were washed with phosphate buffered saline (PBS). Tissues were then transferred to a hypotonic buffer (10×10^{-3} M Tris, 5×10^{-3}M ethylenediaminete-traacetic acid (EDTA), and 1×10^{-6}M phenylmethanesulfonyl-fluoride, pH 8.0) for 1 hour. Next, tissues were frozen and thawed three times in the hypotonic buffer. Tissues were washed gradually with 70% (v/v) ethanol and 100% ethanol for 30 minutes each. Lipids were extracted by 3x30 minutes washes of 100% acetone, followed by a 24-hour incubation in a 60/40 (v/v) hexane: acetone solution (solution was changed three times over 24 hours). The defatted tissue was washed in 100% ethanol for 30 min and incubated overnight at 4°C in 70% ethanol. Next, the tissue was washed four times with PBS (pH 7.4) and incubated in 0.25% Trypsin-EDTA solution

(Biological Industries) overnight. The tissue was washed thoroughly with PBS and incubated in 1.5M NaCl (solution was changed three times in 24 hours), followed by washing in 50 × 10^{-3}M Tris (pH 8.0), 1% triton-X100 (Sigma-Aldrich) solution for 1 hour. The decellularized tissue was washed in PBS followed by double distilled water and then frozen (–20°C) and lyophilized. The dry, decellularized omentum was ground into powder (Wiley Mini-Mill, Thomas Scientific, Swedesboro, NJ). The milled omentum was then enzymatically digested for 96 hours at room temperature under stirring, in a 1 mg mL^{-1} solution of pepsin (Sigma-Aldrich, 4000 U mg^{-1}) in 0.1M HCl. Subsequently, the pH was adjusted to 7.4 using 5M NaOH and DMEM/F12 × 10 (Biological industries). The final concentration of decellularized omentum in the titrated solution was 1% (w/v).

Rheological properties. Rheological measurements (n=6) were performed using a Discovery HR-3 hybrid Rheometer (TA Instruments, DE) with 8 mm diameter parallel plate geometry and a Peltier plate to maintain the sample temperature. Samples were prepared by encapsulation of AuNPs at different concentrations (50 and 0.5 µg mL^{-1} for high and low concentrations, respectively) or medium for control, to reach a final hydrogel percentage of 0.6% and 0.2%. In order to examine the solidification process, samples were loaded at a temperature of 4°C, which was then raised to 37°C, during which the oscillatory moduli of samples were monitored at a fixed frequency of 0.8 rad s^{-1} and a strain of 1%.

TEM sections. Samples of AuNPs encapsulated within pristine hydrogel were fixed in 2.5% glutaraldehyde (diluted in PBS) over night at 4°C. After several washings in PBS, samples were fixed in 1% OsO_4 in PBS for 2 hours at 4°C. Dehydration was carried out in graded ethanol followed by embedding in Glycid ether. Thin sections were mounted on Formvar/Carbon coated grids and examined using Jeol 1400 – Plus transmission electron microscope (Jeol, Japan). Images were captured using SIS Megaview III and iTEM (Olympus).

Cardiac cell isolation and seeding. Neonatal cardiac cells were isolated according to Tel Aviv University ethical use protocols from intact ventricles of 1- to 3-day-old neonatal Sprague-Dawley rats as previously reported[2]. Briefly, left ventricles of 0-3 day old neonatal Sprague-Dawley rats were harvested and cells were isolated using 6 cycles (30 min each) of enzyme digestion with collagenase type II (95 U/mL; Worthington, Lakewood, NJ) and pancreatin (0.6 mg/mL; Sigma-Aldrich) in Dulbecco's Modified Eagle Medium (DMEM, ($CaCl_2$•$2H_20$ (1.8 mM), KCl (5.36 mM), $MgSO_4$•$7H_2O$ (0.81 mM), NaCl (0.1M), $NaHCO_3$ (0.44 mM), NaH_2PO_4 (0.9 mM)). After each round of digestion, the cells were centrifuged (600 g, 5 min) and resuspended in M199 culture medium (Biological Industries, Beit-Haemek, Israel)

supplemented with 0.6 mM $CuSO_4 \bullet 5H_2O$, 0.5 mM $ZnSO_4 \bullet 7H_2O$, 1.5 mM vitamin B12, 500 U/mL Penicillin and 100 mg/mL streptomycin, and 0.5% (v/v) FBS. To enrich the cardiomyocytes population, cells were suspended in culture medium with 5% FBS and pre-plated twice (30 min). Next, cells were counted and seeded within the nanocomposite and pristine hydrogel at $2x10^7$ cells in 100 μL pristine hydrogel (in order to achieve a final concentration of 0.6%). For ROS and bioluminescence experiments, cardiac cells were counted and seeded on white cell plates at a concentration of $5x10^5$ cells per mL medium. The cell-seeded implants or plates were cultivated at 37°C in a 5% carbon dioxide humidified incubator.

Cell viability. Implant viability was determined using a live/dead fluorescent staining assay with fluorescein diacetate (7 μg mL^{-1}, Sigma-Aldrich) and propidium Iodide (5 μg mL^{-1}, Sigma-Aldrich) for 20 min at 37°C. Live and dead cells within the different implants were visualized by an inverted fluorescence microscope.

Luminescence assay. All measurements were performed over time using Luminometer (Turnerbiosystems) with time intervals of 5 minutes. Each treatment was performed on triplicates wells. For these measurements, chemiluminescent luminophore at a concentration of 10 mM (0.1% DMSO) was used. In order to assess the *ex vivo* effect of AuNPs on ROS levels, AuNPs at different concentrations (5, 0.5, 0.05 and 0.005 mg mL^{-1}) were incubated with 30% H_2O_2 (100 μM, Bio-Lab), and ROS levels were measured. As a control, wells without the addition of AuNPs and H_2O_2 were tested. For cell experiments, cardiomyocytes were incubated for 2 hours with AuNPs at different concentrations (0.5, 0.05 and 0.005 mg mL^{-1}) under mild shaking in 37°C. Following 3 washes with PBS and incubation with the probe, luminescence levels were measured for 20 minutes before the treatment. Afterwards, intracellular ROS levels were increased by a 30-min incubation with menadione (37°C, 100 μM, Sigma-Aldrich) and measured for up to 1-hour post treatment. For implant experiments, AuNPs at different concentrations (0.5, 0.05 and 0.005 mg mL^{-1}) were encapsulated within pristine hydrogel and cardiomyocytes ($6x10^6$ cells per implant) to reach a final hydrogel concentration of 0.6%. For control implants, pristine hydrogel was diluted with medium at the same ratio as AuNPs. After solidification at 37°C and incubation with the probe, intracellular ROS levels in the implants were increased by menadione and measured over time. In order to evaluate the effect of the composite hydrogel on intracellular ROS, we first tested the effect of pristine hydrogel. For that, pristine hydrogel and alginate gel (5/60, FMC) at the same concentrations (0.6, 0.4 and 0.2%) were added to

plate wells to form a thin layer covering the cells. Next, PBS, alginate gel, pristine hydrogel, and composite hydrogels containing different concentrations of AuNPs (0.5, 0.05 and 0.005 mg mL^{-1}) were added. All hydrogel samples were in their liquid form and at a final concentration of 0.25% obtained by mixing 100 µl 1% pristine hydrogel with 300 µl AuNPs or medium solution.

Animal study. All mice were treated according to ethical regulations of Tel Aviv University. Permission was granted by the Institutional Animal Care and Use Committee (IACUC) of Tel Aviv University, protocol number 04-20-012- “Treatment of biological hydrogel and cardiac cells on cardiac regeneration after ischemia reperfusion injury in mice model”.

MRI imaging. C57BL/6 male mice, weighing from 24 to 32 g, were examined before, 1 and 6 weeks post the IRI. All experimental procedures were performed under general anesthesia induced by 1-1.5% isoflurane in pure oxygen. MRI imaging was performed using a 7T/30 MRI Biospec (Bruker BioSpin, Germany) equipped with a gradient unit of 660 mT m^{-1}. MRI scans were acquired using a cross coil setup including 86 mm resonator and a 20 mm array of surface coil. The body temperature of the animals was maintained by circulating warm water and all MRI scans were performed with simultaneous ECG and respiration gating. Mice were placed in the magnet with the ears positioned at the isocenter.

In order to identify the morphology of the heart tissue, Cine-FLASH (fast low angle shot) MRI was used to image the heart in a short-axis and long-axis view. Six short-axis slices covering the heart from the base to the apex and one long-axis slice were obtained to evaluate myocardial function and infarct size. The following sequence parameters were used: repetition time (TR) = 4.5 ms; echo time (TE) = 2.3 ms; flip angle (FA) = 10°; field of view (FOV) = 25x25 mm^2; matrix size = 192x192; slice thickness = 1 mm. Cine movies with 10 frames per slice were reconstructed retrospectively. Each Cine scan lasted 2 minutes.

Implant and heart immunostaining. Implants were fixed in 4% formaldehyde for 20 minutes, permeabilized with 0.1% (v/v) triton X-100 for 10 minutes and blocked with 5% bovine serum albumin in PBS for 1 hour. Then, implants were stained with primary antibodies in 0.5% blocking solution for 2 hours. α-sarcomeric actinin (1:200, ab9465, Abcam) and α-connexin 43 (1:1000, ab11370, Abcam) were used as primary antibodies. For heart sections immunostaining, primary antibodies for troponin (1:100, ab47003, Abcam) and collagen (1:4000, MA1-26771, Thermo Fisher) were used. After 3 washes with 0.5% blocking solution, implants were incubated with Alexa Fluor 488-conjugated goat anti-rabbit antibody and Alexa Fluor 647-conjugated goat anti-mouse antibody (1:250; Jackson, West

Grove, PA) in 0.5% blocking solution for 2 hours. For nuclei detection, implants were incubated for 5 minutes with Hoechst 33258 (1:100; Sigma-Aldrich) in PBS and washed 3 times. Samples were imaged using a confocal microscope (Nikon Eclipse NI-E). Images were processed and analyzed using NIS elements software (Nikon Instruments).

Heart immunohistochemistry. Heart sections were also stained for blood vessels detection. Sections were fixed, permeabilized and blocked as specified above. Then, the sections were stained with α-CD-31 (100 µg, ab124432, Abcam) primary antibodies in blocking solution for 2 hours. After 3 PBS washes, sections were incubated with Alexa Fluor 488-conjugated goat anti-rabbit antibody (1:250; Jackson, West Grove, PA) in blocking solution for 2 hours. For macrophages and leukocytes staining, the sections were stained with PE anti-mouse CD11b (1:250, Biolegend) and Alexa Fluor 488 anti-mouse CD45 (1:250, Biolegend). For MHC class I staining, the sections were stained with anti-MHC class I antibody [OX18] (1:1000, ab6405, abcam) which was labeled with cy5 (Cy5® Conjugation Kit (Fast) - Lightning-Link®, ab188288, abcam). For nuclei detection, sections were incubated for 5 minutes with Hoechst 33258 (1:100; Sigma-Aldrich) in PBS and washed 3 times. All samples were imaged using a confocal microscope (Nikon Eclipse NI-E). Images were processed and analyzed using NIS elements software (Nikon Instruments).

Supporting Figures

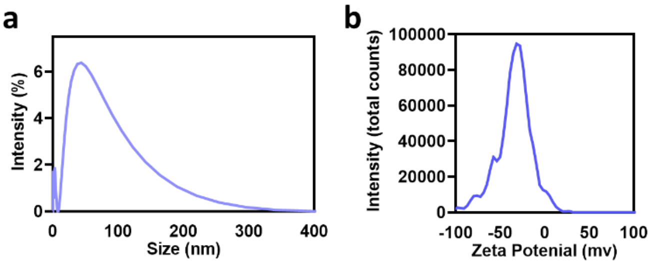

Z average (nm)	Zeta Potential (mV)
24.3±3.4	-38.5±0.2

Figure S1: AuNPs properties. (a) DLS. (b) Zeta potential measurements

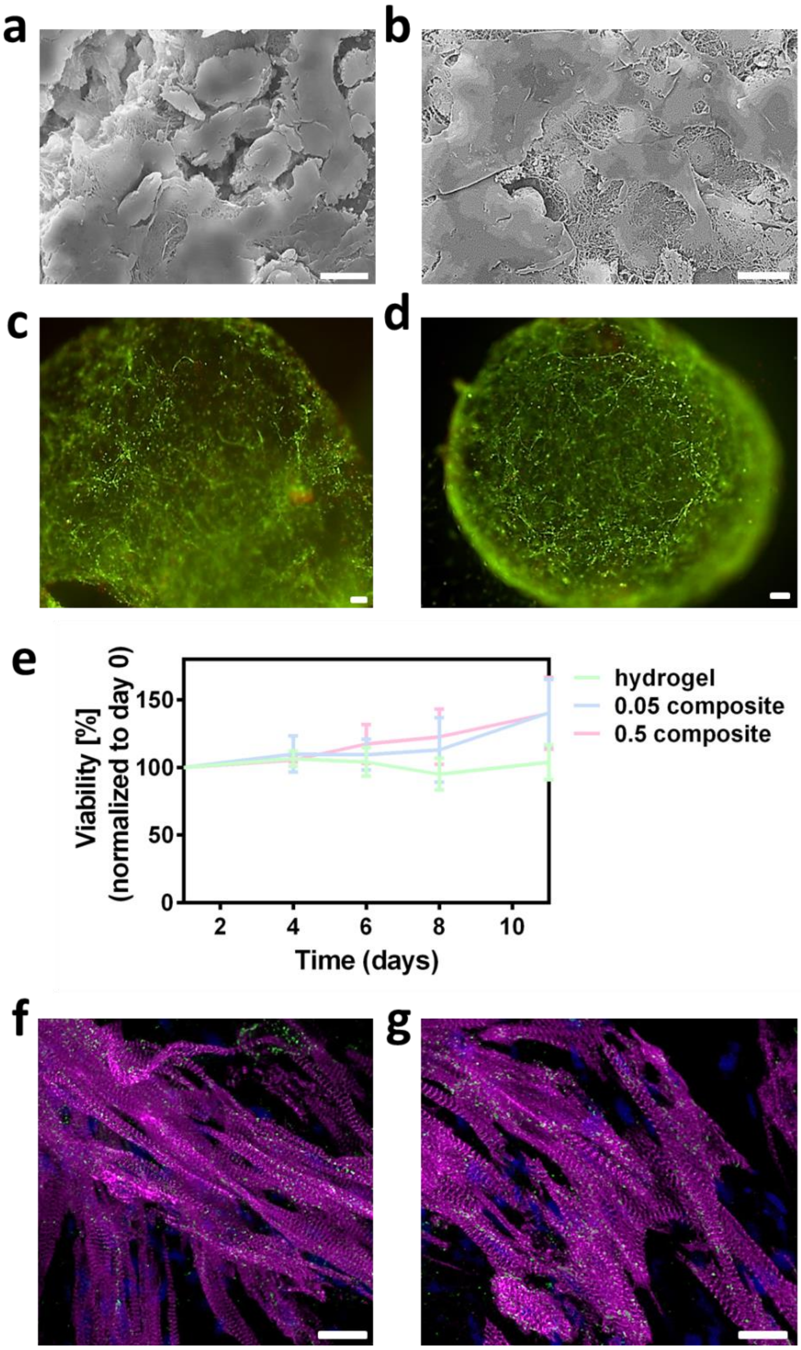

Figure S2: Cardiac implants morphology, viability and organization. SEM images of the cardiac cells within the (a) pristine and (b) high AuNPs concentration composite hydrogel, bar scale 10 µm. (c, d) Viability of the cells encapsulated within (c) pristine and (d) high AuNPs concentration composite implants tested 1 week post cell encapsulation by live/dead analysis (live cells in green and dead cells in red), bar scale 100 µm. (e) Viability of the cells encapsulated within pristine, low and high AuNPs concentration composite implants tested up to 10 days post cell encapsulation by presto blue analysis. (f, g) Cardiac sarcomeric actinin immunostaining on day 14 post cells encapsulation of (f) pristine hydrogel and (g) high AuNPs concentration composite hydrogel. Actinin is in pink, connexin 43 in green and nuclei in blue, scale bar 20 µm.

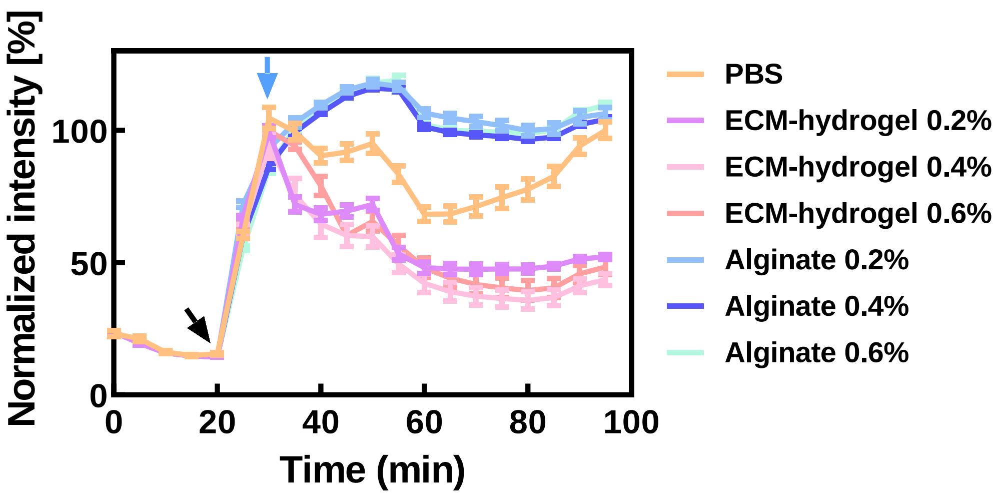

Figure S3: Normalized intensity of ROS levels post menadione addition (black arrow) to different concentrations of alginate and pristine hydrogel (blue arrow). As a control, PBS was added to the medium.

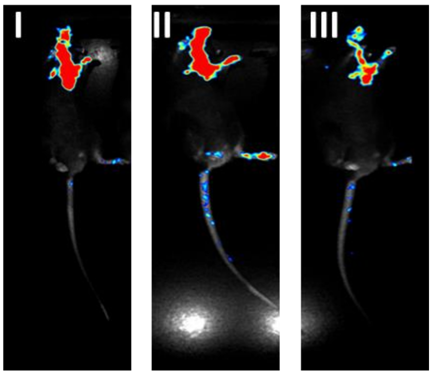

Figure S4: Bio-luminescence imaging of (I) untreated, (II) pristine hydrogel- and (III) composite hydrogel-treated mice, 1-week post-IRI.

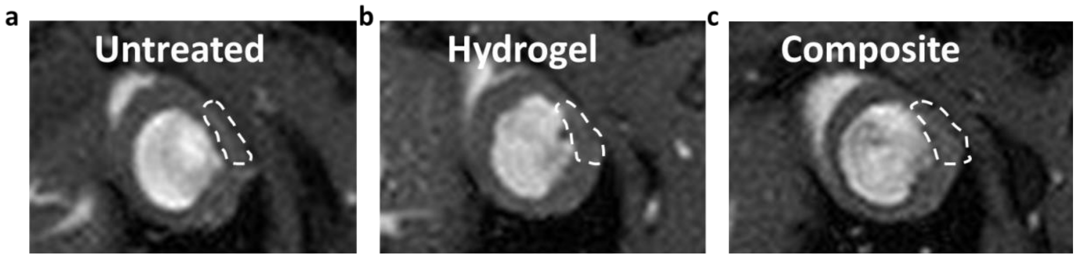

Figure S5: Representative MRI images for the different treatments, 6-weeks post-IRI. White dashed areas indicate the injured area.

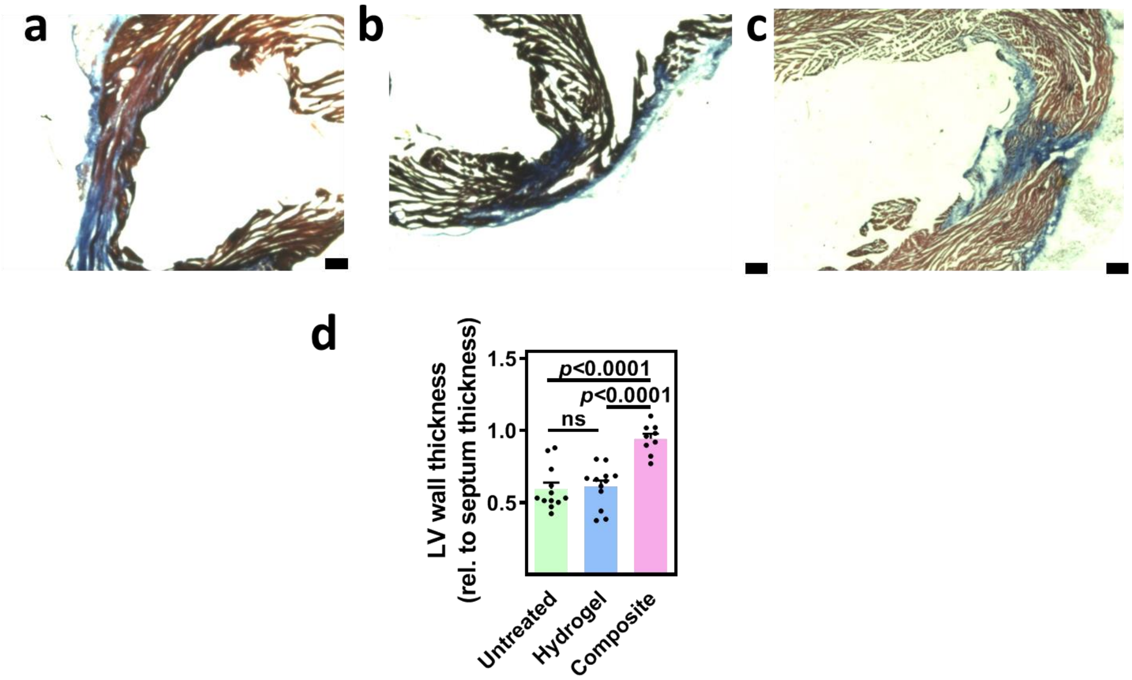

Figure S6: Masson Trichrome staining of (a) untreated, (b) pristine hydrogel- and (c) composite hydrogel treated mice, 6-weeks post-IRI. Scale bar 500 µm. (d) LV wall thickness relative to the septum thickness for all treatments.

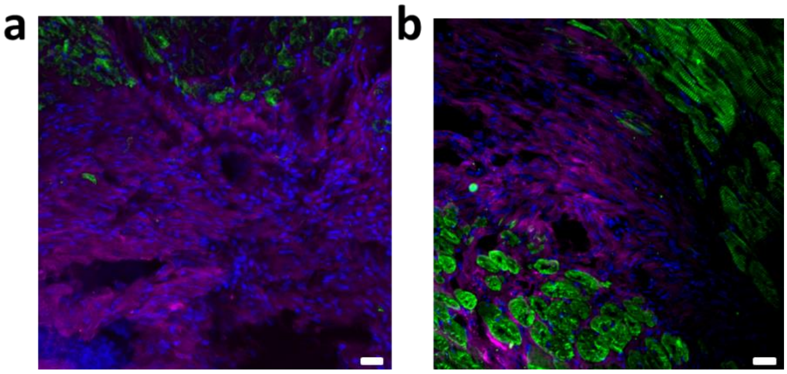

Figure S7: Fluorescent immunostaining of the scar tissue 6 weeks post IRI, for (a) untreated and (b) pristine hydrogel treatment. Cardiac troponin is in green, collagen in pink and nuclei in blue, scale bar 20 µm.

a b c

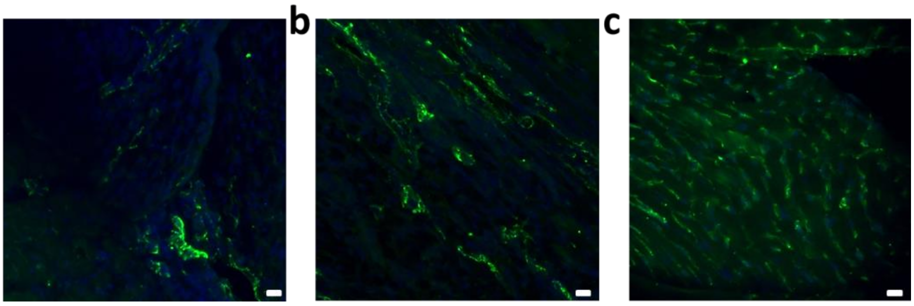

Figure S8: Fluorescent immunostaining of blood vessels within the scar tissue of (a) untreated, (b) pristine hydrogel- and (c) composite hydrogel- treated mice. CD31 is in green and nuclei in blue, scale bar 10 µm.

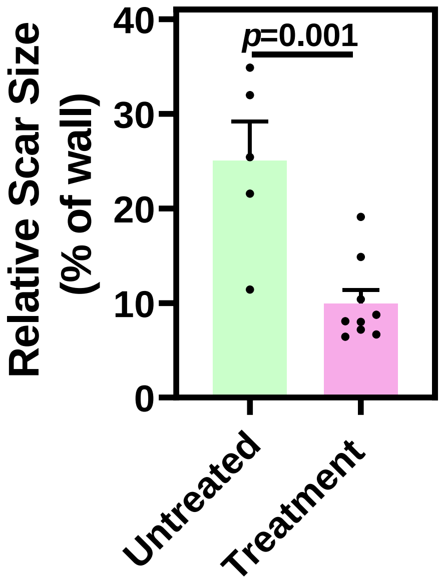

Figure S9: Relative scar size as indicated by Masson Trichrome histology for treated and untreated mice.

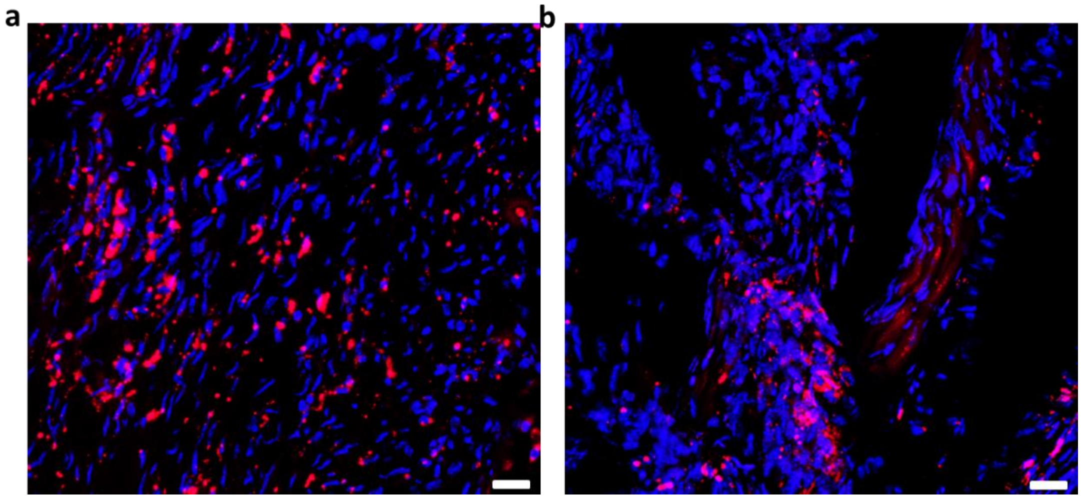

Figure S10: Fluorescent immunostaining of macrophages within the scar tissue of (a) untreated and (b) treated mice. CD11b is in red and nuclei in blue, scale bar 20 µm.

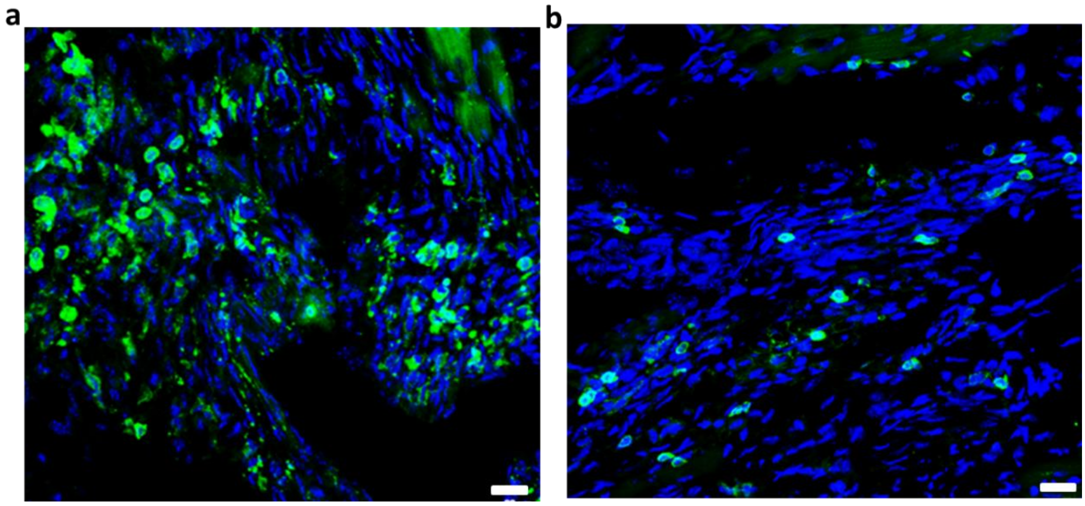

Figure S11: Fluorescent immunostaining of leukocytes within the scar tissue of (a) untreated and (b) treated mice. CD45 is in green and nuclei in blue, scale bar 20 µm.

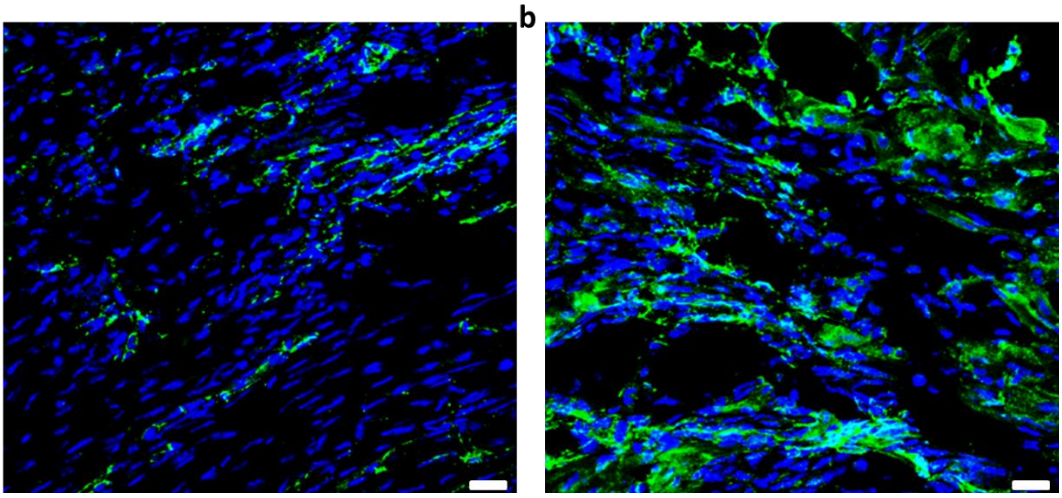

a b

Figure S12: Fluorescent immunostaining of CD31 positive cells within the scar tissue of (a) untreated and (b) treated mice. CD31 is in green and nuclei in blue, scale bar 20 µm

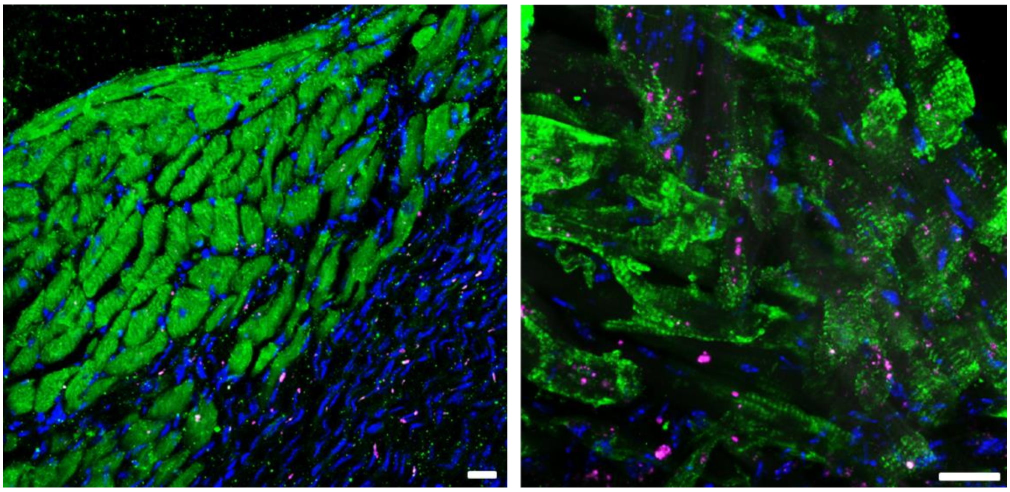

Figure S13: Fluorescent immunostaining of MHC class I and troponin within the myocardium of treated mice. MHC class I is in pink, cardiac troponin is in green and nuclei in blue, scale bar 20 µm

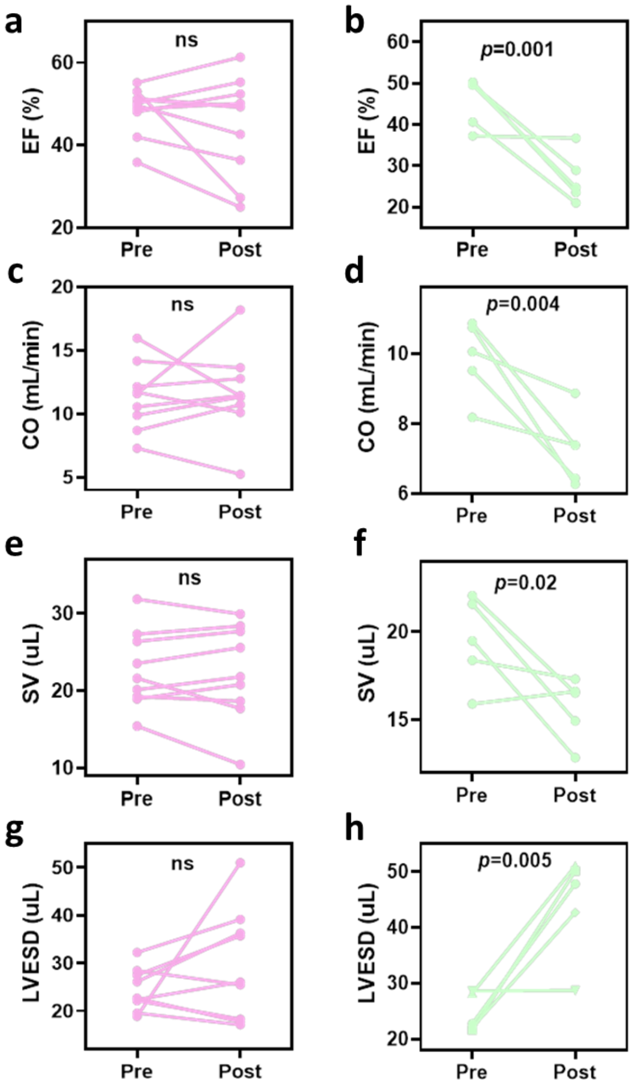

Figure S14: Echocardiographic individual analysis of ejection fraction (EF) (a,b), cardiac output (CO) (c,d), stroke volume (SV) (e,f) and LV end-systolic volume (LVESV) (g,h) pre and post-IRI surgery for treated (a,c,e,g) and untreated mice (b,d,f,h).

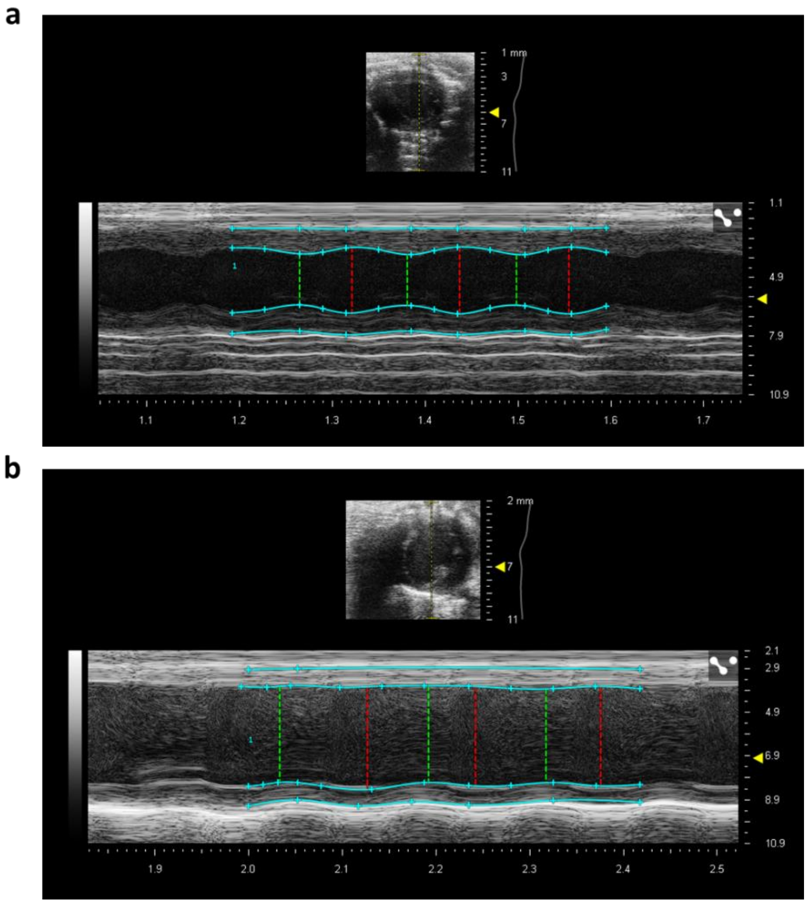

Figure S15: Representative echocardiographic images of M-mode for treated (a) and untreated (b) mice.

References

1. Edri, R. et al. Personalized hydrogels for engineering diverse fully autologous tissue implants. *Advanced materials* **31**, 1803895 (2019).
2. Dvir, T., Benishti, N., Shachar, M. & Cohen, S. A novel perfusion bioreactor providing a homogenous milieu for tissue regeneration. *Tissue engineering* **12**, 2843-2852 (2006).
